# Paradigmatic enhancement of stem vowels in regular English inflected verb forms

**DOI:** 10.1007/s11525-021-09374-w

**Published:** 2021-02-11

**Authors:** Fabian Tomaschek, Benjamin V. Tucker, Michael Ramscar, R. Harald Baayen

**Affiliations:** 1 Seminar für Sprachwissenschaft, Universität Tübingen, Wilhelmstraße 19-23, 72074 Tübingen, Germany; 2 Department of Linguistics, University of Alberta, 4-32 Assiniboia Hall, Edmonton, Alberta T6G 2E7 Canada

**Keywords:** Speech production, Articulography, Phonetic enhancement, Quantile GAMMs, Inflection, Paradigmatic probability

## Abstract

Many theories of word structure in linguistics and morphological processing in cognitive psychology are grounded in a compositional perspective on the (mental) lexicon in which complex words are built up during speech production from sublexical elements such as morphemes, stems, and exponents. When combined with the hypothesis that storage in the lexicon is restricted to the irregular, the prediction follows that properties specific to regular inflected words cannot co-determine the phonetic realization of these inflected words. This study shows that the stem vowels of regular English inflected verb forms that are more frequent in their paradigm are produced with more enhanced articulatory gestures in the midsaggital plane, challenging compositional models of lexical processing. The effect of paradigmatic probability dovetails well with the *Paradigmatic Enhancement Hypothesis* and is consistent with a growing body of research indicating that the whole is more than its parts.

## Introduction

Many theories of word structure in linguistics and morphological processing in cognitive psychology are grounded in a compositional perspective on the (mental) lexicon in which complex words are composed during speech production from (and decomposed during comprehension into) sublexical elements such as morphemes, stems, and exponents (see Ramscar and Port 2016, for a critical review). When combined with the hypothesis that storage in the lexicon is restricted to irregular words, the prediction follows that properties specific to regular inflected words should not co-determine the phonetic realization of these inflected words. This study shows that the stem vowels of more frequent regular English inflected verb forms are produced with more enhanced articulatory gestures in the midsaggital plane, challenging strictly compositional models of the lexicon and lexical processing.

In what follows, we first provide an overview of some influential models of lexical processing and the role of decomposition and frequency of occurrence in these models. Subsequently, we discuss the challenges for compositional theories posed by a growing body of literature that documents how the phonetic detail of regular complex words is to some extent predictable from whole-word properties, including whole-word frequency.

The core of this study is a production experiment using electromagnetic articulography to study tongue movements in the midsaggital plane for two stem vowels in English regular inflected verb forms. The central question addressed by this experiment is whether the experience that speakers have with the inflected verb form itself is predictive for how the tongue’s articulatory gestures are executed. Anticipating the results, we observed articulatory enhancement for words that are more frequent within their inflectional paradigm, a result that dovetails well with the *Paradigmatic Enhancement Hypothesis* (Kuperman et al. 2007). In the General Discussion, we reflect on the implications of these findings, which are consistent with a growing body of research reporting that the whole is more than its parts, for both linguistic and cognitive theories of the lexicon.

## Models of word production

Formal theories of word structure seek to provide a characterization of lexical knowledge that is maximally parsimonious, while at the same time providing correct predictions about what word forms are possible. Several theoretical frameworks model the morphological system as a lexical calculus, which combines a set of basic units (stems, affixes, morphemes) with a set of rules for assembling these units into well-formed combinations. These frameworks differ with respect to the nature of the units. *Item-and-Arrangment* theories (Hockett 1954) assume that these units are Saussurian signs (Saussure 1916). *Paradigm Function Morphology* (Stump 2001; Bonami and Stump 2016) works with stems and formatives (exponents) as basic units of form, and provides rule systems that regulate how bundles of semantic features are expressed in combinations of stems and exponents. The mapping between form primitives and semantic primitives is no longer constrained to be one-on-one: Multiple semantic features can be realized in one exponent, and one semantic feature can be realized in multiple exponents. *Distributed Morphology* (Halle and Marantz 1994; Marantz 2013), which merges morphology into syntax, is also a realizational theory. Vocabulary insertion rules specify how to assemble stems and exponents given sets of inflectional and syntactic features at terminal nodes of syntactic trees.

A completely different perspective is presented by *Word and Paradigm Morphology* that takes whole words to be the basic unit of morphological analysis (Matthews 1991). Stems and exponents have no theoretical status, but are useful for describing proportional analogies within paradigms. These analogies are assumed to drive inflectional productivity. Inspired by *Discriminative Learning* (Ramscar and Yarlett 2007; Ramscar et al. 2010, 2013a,b; Baayen et al. 2011), a computational model that implements *Word and Paradigm Morphology* was proposed in Baayen et al. (2019), under the name of the *Discriminative Lexicon*. The *Discriminative Lexicon* does not work with lexical representations for form and meaning that are stored in some list-like dictionary. The networks of the *Discriminative Lexicon* are its memory. A word’s meaning is generated on the fly from visual or acoustic input, and a word’s form is generated on the fly given the message the speaker is seeking to encode, without requiring constructs such as morphemes, stems, and exponents (Baayen et al. 2018b; Chuang et al. 2020).

These various models of the lexicon differ with respect to how they view the relation between morphological theory and the neural architectures subserving lexical processing, on the one hand, and lexical processing and phonetic realization on the other hand. *Item-and-Arrangement* (Hockett 1954), *Word and Paradigm Morphology* (Matthews 1991), and *Paradigm Function Morphology* (Stump 2001; Bonami and Stump 2016) do not make any claims about whether their algorithmic structure might provide a blueprint of cognitive mechanisms, and consequently how they shape phonetic realizations.

The version of *Word and Paradigm Morphology* developed by Blevins (2016) argues that only words have cognitive reality, maintaining that stems and exponents are useful descriptive devices only. The *Discriminative Lexicon* (Baayen et al. 2019) is a theory that goes a step further. It purports to provide a blueprint of the mental lexicon. Since the simple networks that it works with are far removed from real neural networks, this blueprint is a functional one. The networks serve as a mathematical tool for predicting words forms and meanings, the cognitive costs of lexical processing in comprehension and production, and the fine phonetic detail with which words are produced.

Among formal theories, *Distributed Morphology* also claims to provide a blueprint for the neural architecture of lexical processing (Pylkkänen et al. 2004; Solomyak and Marantz 2010; Marantz 2013; Pinker 1999), and the same holds for the dual mechanism model of Pinker (1997). One claim made by *Distributed Morphology* and the Dual Mechanism model is that regular complex words do not have representations in the mental lexicon. The mental lexicon posited by these theories is maximally parsimonious, allowing as basic units of the morphological calculus only stems, exponents, and irregular complex words. These theories rule out that the frequency with which regular complex words are used would be predictive for how they are processed and articulated. It is noteworthy that this prediction does not follow from linguistic theories themselves. For instance, Jackendoff (1975) has argued that regular complex words are stored, but that within a linguistic evaluation metric for parsimony, this storage does not make the grammar more complex. One way in which this insight can be re-conceptualized is that under a good compression scheme, the costs of storing regular complex forms is much smaller than is the case in a lexicon without compression. Complex words can be added to a lexical inventory with minimal additional storage requirements, precisely because they are so predictable (see also Juola 1998).

The insight of Jackendoff (1975) has not been taken up by mainstream psycholinguistics. Psycholinguistic models (e.g. Rastle et al. 2004; Smolka et al. 2009) have mostly adopted constituent-based approaches to lexical processing that sometimes resemble realizational theories and sometimes are closer to item-and-arrangement models. Accordingly, psycholinguistic models adopt the principle of parsimony that rules out representations in the mental lexicon for regular complex words. Hence, these theories, like *Distributed Morphology* and the *Dual Mechanism Model*, also predict that the frequency with which regular complex words are used is irrelevant for predicting how such words are produced.

Consider, for instance, one influential model of speech production, the *WEAVER++* model (Roelofs 1997b; Levelt et al. 1999). Content words are coupled with one or more lemma representations, which are abstract place holders for dictionary entries, which are linked to inflectional feature nodes. The constellation of a lemma and its active inflectional nodes jointly drive the selection of stems and exponents at the form level. These stems and exponents in turn activate phone units, which subsequently are bundled into syllables. Syllables constitute the input for articulatory motor programs, such as those proposed by Browman and Goldstein (1986), Guenther (1995), Turk and Shattuck-Hufnagel (2020). Importantly, in this modular feed-forward system, the selection and activation of lower-level units is driven entirely by the units found one level up in the hierarchy. *WEAVER++* allows frequency of occurrence to play a role at two stages of the model, lemma access and syllable access (Jescheniak and Levelt 1994; Schriefers et al. 1990; Levelt et al. 1991; Roelofs 1997a; Cholin et al. 2004, 2006). The architecture of *WEAVER++* is explicitly designed in such a way that neighborhood density and the frequencies with which complex words occur cannot co-determine articulation.

The *interactive activation model* of Dell (1986) shares with *WEAVER++* a hierarchy of representational levels and processes operating on these levels. The way in which this model is set up appears to be somewhat closer to *item-and-arrangement morphology*. For instance, semantic units for inflectional functions such as plurality are linked up with the corresponding form units. The model does not incorporate semantic units or form units for complex words, and hence it does not predict effects of whole-word frequency. A related model using the mechanism of interactive activation is the *semantic-phonological model* (Foygel and Dell 2000; Dell 1990; Dell et al. 1997; Schwartz and Brecher 2000; Schwartz et al. 2006; Dell et al. 2007), which works with a semantic layer, a word form layer, and a phonological layer. In order to model the word frequency effect, the model assigns different association strengths to the connections between units, across all three levels (Kittredge et al. 2008). The model has not addressed the production of morphologically complex words, and whole-word frequency effects are unlikely to be within the scope of the model as long as whole-word semantic and form representations are not added to the model’s inventory of representations.

Production models that incorporate learning—such as the *past-tense model* (Rumelhart and McClelland 1986), *Discriminative Learning* models of plural production (Ramscar and Yarlett 2007; Ramscar et al. 2010, 2013b), the *multi-layer back-propagation model* (Mirkovic et al. 2011), and the *Discriminative Lexicon* model (Baayen et al. 2019; Chuang et al. 2020)—are inherently sensitive to the frequency with which they encounter words in their input. As models are trained incrementally, network weights will become honed towards words that occur more often in the training data. However, the performance of these models does not only depend on the frequency of occurrence of words during learning, but also, critically, on the similarity structure of their forms and meanings, and the frequencies of words similar in form and meaning that are encountered during learning.

## The phonetic realization of complex words

Linguistic theories of morphology, as well as compositional models of speech production in psychology, produce phonological representations at their output level. These representations typically consist of a sequence of syllables. At the suprasegmental level, sequences of syllables can be grouped into feet and at the segmental level, they can be further broken down into onsets and rimes. Following the hypothesis of the *dual articulation of language* (Martinet 1965), the way that sounds and syllables are organized follows its own rules. These rules operate independently of words’ meanings and the stems and exponents that were used to assemble the sounds, syllables, and their ordering in time. Furthermore, all compositional models share the assumption that words have canonical, abstract, phonological representations. Even though actual phonetic realizations may diverge from canonical representations, it is assumed that these actual phonetic realizations can be adequately and completely handled by phonetic rules that operate on the phonological representations.

### Phonetic variation and morphological complexity

One problem that models with this strict division of labor run into is that how words are actually pronounced can differ substantially from canonical phonological representations in unpredictable ways (see, e.g. Johnson 2004; Ernestus et al. 2002; Kemps et al. 2004). Not only is it the case that many reduced forms cannot simply be derived by rule from the corresponding canonical forms (Ernestus 2000), but also the variability in the production of individual words increases as their frequency increases (Linke and Ramscar 2020).

Importantly, many ‘aberrant’ phonetic variants typically express a wide range of pragmatic features that are an intrinsic part of the message (Hawkins 2003). This holds not only for monomorphemic words, but also for complex words (Keune et al. 2005). Hanique and Ernestus (2012) reported that phonetic reduction in morphologically complex words was consistently predicted by the frequency of the whole word rather than by measures tied to constituents, a result that is consistent with recent findings for language comprehension (Giraudo and Orihuela 2015; Schmidtke et al. 2017).

A further problem for classical compositional models is that, over the last two decades, substantial evidence has been accumulating that the phonetic detail with which complex words are realized can be highly specific to their meaning and morphological status. Using electromagnetic articulography, Cho (2001) observed that the variability in gestural coordination during the articulation of consonant clusters was larger when the consonants were located at morpheme boundaries than when they were within a morpheme (see also Gafos et al. (2010) for similar results in terms of gestural overlap). Using articulography and acoustic data, Lee-Kim et al. (2012) have found that the “darkness” of English [l] depends on its morphological status. Also using electromagnetic articulography, Tomaschek et al. (2018a) observed that morphologically complex words have faster articulations in complex articulatory movements when the whole word was more frequent. Complex words that are more frequent also have smoother articulatory transitions between subsequent gestures (Tomaschek et al. 2013). Tomaschek et al. (2018c) put forward the hypothesis that the motor skills required for pronouncing a complex word benefit from practice, a proposal that dovetails well with other findings in kinematic studies of articulation (Tiede et al. 2011; Tomaschek et al. 2020) and hand movements (Sosnik et al. 2004).

The acoustic duration with which complex words, or parts thereof, are realized, has also been a highly informative variable. Drager (2011) and Podlubny et al. (2015) observed that segment durations in the English word ‘like’ depended on its specific grammatical function. Word frequency also emerged as one of the determinants of the acoustic duration of homophones (Gahl 2008; Lohmann 2018). Plag et al. (2017) and Seyfarth et al. (2018) extended the evidence from morphologically simple to morphologically complex words by studying the acoustic duration of the supposedly homophonous /s/ suffix of English, which realizes a range of inflectional functions (plural on nouns, singular on verbs, genitive singular, genitive plural, as well as reduced auxiliaries). Even though their results seem to be contradictory, they reported systematic differences in central tendency across the different morphosyntactic functions of the /s/.

### Frequency of occurrence and phonetic realization

Although the frequency with which complex words are used is well established as a factor co-determining lexical processing time in comprehension (Frauenfelder and Schreuder 1992; Baayen et al. 1997, 2003, 2008; Schmidtke et al. 2017, 2018), it is still under debate what role whole-word frequency plays in speech production. Picture naming experiments on Dutch noun singulars and plurals appear to support the architecture of the *WEAVER++* model (Levelt et al. 1999). However, the observed pattern of results can also be understood as a paradigmatic effect (Baayen et al. 2008). Furthermore, several other studies have observed effects of whole-word frequency in chronometric tasks (see, e.g., Bien et al. 2005, 2011; Janssen et al. 2008).

Research on reaction times has been complemented by investigations addressing the properties of the speech produced, with acoustic duration as the primary variable of interest. In this complementary line of research, frequencies are typically recast as probabilities. Two kinds of probabilities can be distinguished: syntagmatic probabilities, which condition on the preceding or following context, and paradigmatic probabilities, which fix contexts and consider the probabilities of competing alternative realizations. These two kinds of probabilities have been found to be predictive for a range of acoustic measures, including words’ acoustic durations and degree of vowel centralization.

According to the *Smooth Signal Redundancy Hypothesis* (Aylett and Turk 2004, 2006), words that are syntagmatically more predictable are less informative and more redundant, thus phonetically reduced (see also Cohen Priva 2015; Schulz et al. 2016; Hall et al. 2018; Brandt et al. 2019; Le Maguer et al. 2016; Priva and Jaeger 2018; Jaeger 2010; Malisz et al. 2018). Since high frequency words are also syntagmatically more predictable, it follows that high frequency words are more redundant than low frequency words. The *Smooth Signal Redundancy Hypothesis* argues that the phonetic reduction associated with syntagmatic predictability is the result of a cognitive constraint requiring that the variance in the amount of information conveyed per unit of time in the speech signal is minimized. According to Aylett and Turk, reduction and enhancement are mediated by prosodic prominence. A more general account that avoids mediation via prosody is presented by (Jaeger 2010)’s *Uniform Information Density Hypothesis* (see also Frank and Jaeger 2008).

With respect to the paradigmatic dimension, a series of studies has revealed effects that go in the opposite direction. Kuperman et al. (2007) observed that interfixes with greater paradigmatic probability were realized with longer durations. This study formulated a hypothesis, the *Paradigmatic Enhancement Hypothesis* (Kuperman et al. 2007). The hypothesis states that phonetic contrasts are enhanced when a word’s probability is higher within the context of its morphological paradigm. The *Paradigmatic Enhancement Hypothesis* has received further support from several other studies. Lõo et al. (2018) found longer acoustic durations of words when they are part of a smaller inflectional paradigmatic family, i.e. when the a-priori probability of an individual word is higher. Bell et al. (2019) reported longer durations for consonants located at the internal word boundary of two-constituent nominal compounds when the family size of the first noun was smaller. Enhancement under increased paradigmatic probability and reduced paradigmatic uncertainty has also been observed for the duration of third person singular [s] in English (Cohen 2014b), the duration of word final [s] in English (Tomaschek et al. 2019), the duration of English stem vowels in regular and irregular verbs (Tucker et al. 2019), and the position of vowels in the vowel space in Russian words (Cohen 2014a, 2015). Tucker et al. (2019) argue that “under increased [paradigmatic] uncertainty, less energy is invested in maintaining duration. Increasing duration would be disadvantageous for the speaker, as the speaker would have to maintain for a longer time a signal that is difficult to discriminate, thus increasing uncertainty in the production process. A longer duration would also be disadvantageous for the listener, as the listener would be confronted for a longer period of time with an ineffective signal that fails to properly reduce the listener’s uncertainty about the message encoded in the speech signal.” (See also Sims (2016).)

In what follows, we further pursue the question of how paradigmatic probability co-determines speech production, by means of an experiment using electromagnetic articulography. We studied the production of English inflected verb forms, such as *walk*, *walks*, *walked*, and *walking*, with the aim of clarifying whether the details of the articulatory trajectory of the tongue when articulating the vowel is predictable from the frequencies with which the different inflected forms are used. The *Paradigmatic Enhancement Hypothesis* suggests that inflected forms that have a higher paradigmatic probability will be articulated with less reduction. Furthermore, from the perspective of motor practice (Tomaschek et al. 2018c, 2020), it seems likely that articulatory skills improve with frequency of use. Thus, practice may allow articulators to reach more extreme positions, thus enabling articulations that produce a signal that is clearer for the listener.

## Electromagnetic articulography experiment

We used Electromagnetic articulography to trace the trajectories of two sensors placed on the tongue during the articulation of four different inflected word forms (e.g., *walk*, *walks*, *walked*, *walking*) for each of 52 different verbs. Tongue movement trajectories in the midsaggital plane were analyzed using the quantile generalized additive model, with as hypothesis that tongue movements over time vary systematically with paradigmatic probability, with greater probability affording more skillful articulation.

### Participants

Twenty-five speakers of Canadian and American English (mean age: 29.4, sd: 8.2) were paid to read out loud the stimuli in randomized order. Ethics approval for the experiment was obtained from the Ethics Board of the University of Alberta, Edmonton.

### Materials and design

We selected 57 English verbs, and collected four inflected variants for each verb: the first person present tense form (*stem*, which is identical to the infinitive), the third person singular present tense form (*stem+s*), the past tense form (*stem+d*) and the progressive form (*stem+ing*). All verb forms, apart from the progressive form, were monosyllabic. The selected word materials comprised [i] and [ɑ] as stem vowels, with VC and VCC rimes. For all words with [ɑ], the vowel was followed by the the voiced alveolar approximant [ɹ]. A list of word forms is available in the supplementary materials (https://osf.io/nrjvx/).

Since word forms that were disyllabic in the *stem+s* and *stem+d* conditions (e.g. ‘pleases’) were not used in the current experiment, the number of tokens for each stem varied. Table 1 presents the number of words in the experiment for each of the two vowels across the four morphological classes, together with examples.

**Table 1:** Number of words with [i] and [ɑ] stem vowels, broken down by morphological condition

	stem	stem+d	stem+s	stem+ing	total	examples
[ɑ]	20	15	20	20	75	arm, armed, arms, arming
	37	29	32	35	133	peel, peeled, peels, peeling

To avoid changes in articulatory patterns due to participants repeatedly pronouncing a lemma more than once in the same experiment (see Shields and Balota 1991; Bard et al. 2000; Tomaschek et al. 2020, for effects of repetition on speech production and articulation), word lists were structured according to a Latin square design, with inflectional variants of the same stem occurring in different lists. In the statistical analyses, the factor *tense*, with levels *stem*, *stem+d*, *stem+s*, and *stem+ing*, was used to model potential systematic effects of a word’s inflectional form.

### Recording

**Fig. 1:**
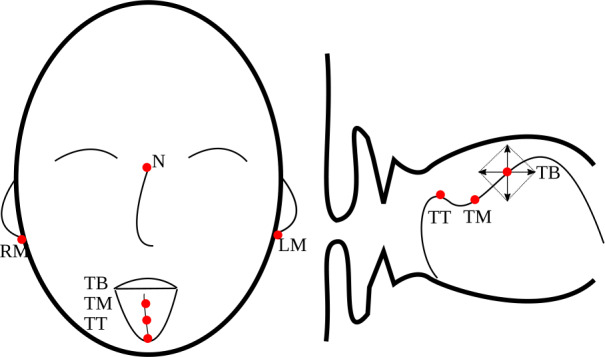
Sensor positions. **Left** frontal illustration. **Right** midsagittal cut through the mouth

Recordings were made in a sound-attenuated booth at the Alberta Phonetics Laboratory in the Department of Linguistics, University of Alberta, Edmonton. Articulatory movements of the tongue were recorded with an NDI wave articulograph at a sampling frequency of 100 Hz. Simultaneously, the audio signal was recorded (Sampling rate: 22.05 kHz, 16 bit) and synchronized with the articulatory recordings. To enable correction for head movements and in order to set up a local coordinate system for tongue movements, a reference sensor was attached to the subjects’ forehead. Before the tongue sensors were attached, a bite plate recording was made to determine the rotation from the local reference coordinate system defined by the magnetic emitter to a standardized coordinate system. On the bite plate, three sensors were attached in a triangular configuration. Tongue movements were captured by three sensors: one slightly behind the tongue tip, one at the tongue middle and one at the tongue body (distance between each sensor: around 2 cm). The present analysis focuses on the tongue tip and the tongue body sensor along both the vertical and the horizontal dimension, which jointly define the midsaggital plain (Fig. 1).

### Preprocessing

Tongue movements were corrected for head-movements in an online procedure during recording by the NDI wave software. The recorded positions of the tongue sensors in the midsaggital plane were centered at the midpoint of the bite plate and rotated in such a way that the back-front direction of the tongue was aligned to the x-axis with more positive values towards the front of the mouth, and more positive z-values towards the top of the oral cavity. Segment boundaries in the audio signal were determined first by automatically aligning the audio signal with words’ phone-based transcriptions by means of P2FA, a Hidden-Markov-Model-based forced aligner for English (Yuan and Liberman 2008). Subsequently, alignments for the vowel were manually verified and corrected where necessary.

The dependent variables in this study were the positions of the tongue tip and tongue body sensors in the midsaggital plane. Absolute sensor positions were transformed into relative distances between the sensor and its maximal vertical/horizontal position for each sensor in each speaker. As a consequence, the z-coordinates represent the (relative) distance from the speaker’s palate. More negative values represent stronger retraction in the horizontal dimension and stronger lowering in the vertical dimension.

Word tokens were realized with variable durations. In order to control for differences in duration and speech rate, time was normalized to the [0, 1] interval, with 0 linked to vowel onset and 1 to vowel offset. In what follows, we refer to this normalized time simply as *time*.

### Predictors

Frequency counts for the verbs and their inflected variants were obtained from an in-house corpus of English movie subtitles containing over 190 million words, collected at https://www.opensubtitles.org (OpenSubtitles 2013). A verb’s lemma frequency $f_{L}$ was defined as the sum of the whole word frequencies $f_{i}$ of its *k* inflected variants:

1 $$ f_{L} = \sum _{i=1}^{k} f_{i}. $$

For the present English verbs, *k* = 4. We defined the paradigmatic probability $p_{i}$ of the *i*-th inflected variant of a verb as its relative frequency in the paradigm:

2 $$ p_{i} = \frac{f_{i}}{f_{L}}. $$

The correlations between the three frequency measures are listed in Table 2.

**Table 2:** Pearson’s product-moment correlations between frequency measures

[i] & [ɑ] vowel	word frequency $f_{i}$	lemma frequency $f_{L}$
lemma frequency $f_{L}$	0.82	
paradigmatic probability $p_{i}$	0.54	0.01

The correlation between lemma frequency and word frequency is too high (0.82) to include both predictors in a regression model. The resulting collinearity is likely to render the interpretation of the statistical model uncertain due to enhancement or suppression (Friedman and Wall 2005; Tomaschek et al. 2018b). The correlation between lemma frequency and paradigmatic probability is close to zero, but for these two predictors, a different problem arises. Frequencies typically follow a Zipfian distribution, and in order to avoid undue effects of outliers, frequencies are log-transformed before being entered as predictors in statistical models (as is the case in the present study). Since $\log (p_{i}) = \frac{f_{i}}{f_{L}} = \log (f_{i}) - \log (f_{L})$, a regression model with both $p_{i}$ and $f_{L}$ as predictors reduces to a model that is linear in $\log f_{i}$ and $\log f_{L}$:

$$\begin{aligned} y =& \beta _{0} + \beta _{1}\log (p_{i}) + \beta _{2}\log (f_{L}) \\ =& \beta _{0} + \beta _{1}[\log (f_{i})-\log (f_{L})] + \beta _{2} \log (f_{L}) \\ =& \beta _{0} + \beta _{1}\log (f_{i}) + [\beta _{2}-\beta _{1}] \log (f_{L}). \end{aligned}$$

In other words, a model including (log-transformed) paradigmatic probability and lemma frequency is equivalent to a model with a whole-word frequency effect and a lemma frequency effect from which the effect of the inflected variant’s frequency has been removed. As a consequence, the interpretation of the predictors and their link to models of lexical processing becomes less straightforward. In the light of these considerations, we decided to fit three models, each one with one of the three frequency measures, and to proceed with the predictor that provides the best fit. For this model, we then also explored whether adding one of the two other frequency measures improves the fit further (in spite of potential problems of collinearity).

We considered including acoustic duration as a covariate. However, linear mixed-effects models regressing acoustic duration on tense, vowel, and the lexical predictors failed to reveal significant effects of paradigmatic probability, frequency and lemma frequency on duration (see the supplementary materials for further details). In order to keep our regression model interpretable, we did not include acoustic duration as covariate. Further modeling (not reported here) clarified that inclusion of duration as predictor does not change the results reported below. We also considered fine-tuning articulatory trajectories for the segmental context of the vowel (using factor smooths, see Tomaschek et al. (2018c) for further details). However, this led to very high concurvity for the present data set. We therefore did not include segmental context as a predictor in the model below, but a model that does so is available in the supplementary materials. Finally, we included speaker and stem as random-effect factors.

### Statistical method

Initially, we attempted to fit a Gaussian generalized additive mixed model (GAMM, Hastie and Tibshirani 1990; Wood 2006, 2011, 2013a,b). GAMM uses spline-based smoothing functions to model nonlinear functional relations between a response and one or more covariates. This enables the analyst to model wiggly curves as well as wiggly (hyper)surfaces (see Wieling et al. 2016; Baayen et al. 2017, for an introduction to spline smooths and their use). However, model criticism revealed that the distribution of the residuals deviated substantially from normality and independence, and resisted any attempts at correction to idd errors. We therefore turned to quantile regression (Koenker 2005), which has recently been integrated with the generalized additive model (Fasiolo et al. 2020). Quantile generalized additive mixed models (QGAMM) provide the analyst with a distribution-free method for estimating the predicted values for any given quantile of the response distribution, together with confidence intervals. In our analyses, we investigated the median, but other quantiles can also be of theoretical interest. We made use of the **qgam** package for R, which builds on the **mgcv** package (version 1.8-3) for R (Version 3.0.3, (Team 2018)). We used the **itsadug** package (van Rij et al. 2015) (Version 2.2) for visualization of the results.

The data points in the present study are not independent: strong temporal autocorrelations are present in the articulatory time series. As a consequence, the model overestimates the amount of independent information, and p-values can be anti-conservative. This anti-conservatism appears to concern primarily the effects for the tongue body in the [i] vowel, as indicated by a Gaussian GAMM with an AR(1) process in the residuals (rho = 0.911). However, in this Gaussian GAMM, the articulatory trajectories are qualitatively very similar to those estimated by the QGAMM. Autocorrelations in the residuals are often also reduced by the inclusion of by-item factor smooths, i.e., non-linear random effects (Baayen et al. 2018a). However, for the present data, including factor smooths results in strong concurvity, which makes it difficult to tease apart what individual predictors contribute to the model and to understand their theoretical significance. To maintain interpretational transparency, we therefore did not include factor smooths. Importantly, prediction accuracy of QGAMMs can by high even in the presence of substantial autocorrelation, as exemplified by the study of Fasiolo et al. (2020) of time series of electricity grid data. As our analyses are exploratory in nature, and given the autocorrelations present in articulatory trajectories, we decided accepting smooth terms in our model as potentially significant only when their associated p-value is less than 0.0001. In other words, we use the QGAMMs as a tool to describe median articulatory positions, and the very small p-values associated with most smooths (≪0.0001) suggest that the model is detecting real signal in the noise.

The trajectories of a tongue sensor in the midsagittal plane, for a given subject and item, can be modeled by an interaction of time by dimension (horizontal vs vertical) by sensor (tongue body vs tongue tip), using treatment coding of factorial predictors. To differentiate between articulatory trajectories for the two vowels ([i], [ɑ]), a further interaction with vowel type needs to be included. Furthermore, since effects can vary by Tense (*stem*, *stem+s*, *stem+d*, *stem+ing*), a further interaction could be added in. However, in order to facilitate modeling and visualization, we did not fit a model with this immense five-way interaction. Instead, we constructed a factor, henceforth SDV, with eight levels, one level for each of the eight combinations of Sensor by Dimension by Vowel. We used QGAMM to fit eight wiggly curves as a function of time, one for each of the levels of SDV. To obtain the four trajectories (for the four combinations of sensor and vowel) as a function of time in the midsaggital plane, the pertinent horizontal and vertical trajectories for a given sensor-vowel combination were combined. The model formula for these curves is: s(Time, by=SDV)

In addition, we fitted eight separate curves, now as a function of one of the lexical predictors word frequency, lemma frequency or paradigmatic probability, with the following specification: s(lexical predictor, by=SDV)

We restricted the effect of Tense to changes in the intercept by including an interaction of SDV and Tense. Adding in by-subject and by-base random intercepts, and including Curves as a fixed-effect factor, we obtained the following model specification: Position ~ SDV + Tense + SDV:Tense +s(Time, by=SDV) + s(lexical measure, by=SDV) +s(Subject, bs="re") + s(Base, bs="re")

This model was fitted to the data for each of the three frequency measures: lemma frequency, word frequency, and paradigmatic probability. A model from which the lexical measure was excluded, Position ~ SDV + Tense + SDV:Tense +s(Time, by=SDV) +s(Subject, bs="re") + s(Base, bs="re")

served as baseline for evaluating the usefulness of the lexical predictor for tongue sensor positions.

## Results

Model comparison of models with the lexical predictors and the baseline model, all fitted with maximum likelihood, was performed with the compareML function from the **itsadug** package. This comparison indicated that paradigmatic probability was most successful at increasing model fit (ΔML = 220.194, Δedf = 16, p < 0.0001), followed by word frequency (ΔML = 205.027, Δedf = 16, p < 0.0001), and at a distance by lemma frequency (ΔML = 7.835, Δedf = 16, p = 0.48).

The goodness of fit of the model with paradigmatic probability can be improved further by adding smooths to the model for word frequency (ΔML = 73.690, Δedf = 16, < 0.0001) or lemma frequency (ΔML = 91.366, Δedf = 16, < 0.0001).^1^ Unfortunately, this results in an unacceptably high degree of concurvity in the model, indicating that hardly any variance can be uniquely attributed to a given frequency measure. In other words, the models with additional frequency measures are overfitting the data. Since qualitatively the effect of paradigmatic probability remains the same in these overly complex models, we have opted for reporting only the model with paradigmatic probability. However, the supplementary materials report the more complex models as well, as well as the models with only word frequency or lemma frequency as (sole) predictor. Results obtained with word frequency are very similar to those obtained with paradigmatic probability (see Appendix). Table 3 presents the summary of the parametric coefficients of the model, and Table 4 presents the summary of the smooths in the model (which also include the random effect factors).

**Table 3:** Summary of parametric coefficients for the model fitted to tongue sensor movements in the midsaggital plane, with **paradigmatic probability** as covariate. SDV = Sensor by Dimension by Vowel, TT = tongue tip, TB = tongue body, ver = vertical movements, hor = horizontal movements

parametric coefficients	estimate	std. error	t-value	p-value
Intercept (TB hor [ɑ], Tense = stem)	−14.6575	0.3091	−47.4163	< 0.0001
Tense = stem+s (TB hor [ɑ])	1.2326	0.2116	5.8264	< 0.0001
Tense = stem+d (TB hor [ɑ])	0.4989	0.2452	2.0347	0.0419
Tense = stem+ing (TB hor [ɑ])	0.3785	0.2557	1.4802	0.1388
SDV = TT hor [i] (Tense = stem)	9.3122	0.2790	33.3766	< 0.0001
SDV = TT hor [i] : Tense = stem+s	−0.9969	0.2252	−4.4260	< 0.0001
SDV = TT hor [i] : Tense = stem+d	−1.1806	0.2607	−4.5293	< 0.0001
SDV = TT hor [i] : Tense = stem+ing	−0.7144	0.2693	−2.6533	0.0080
SDV = TB hor [i] (Tense = stem)	10.6854	0.2795	38.2284	< 0.0001
SDV = TB hor [i] : Tense = stem+s	−1.1241	0.2265	−4.9634	< 0.0001
SDV = TB hor [i] : Tense = stem+d	−0.9085	0.2625	−3.4611	0.0005
SDV = TB hor [i] : Tense = stem+ing	−0.6452	0.2698	−2.3911	0.0168
SDV = TT ver [i] (Tense = stem)	5.2337	0.2804	18.6637	< 0.0001
SDV = TT ver [i] : Tense = stem+s	−0.8055	0.2303	−3.4982	0.0005
SDV = TT ver [i] : Tense = stem+d	0.3809	0.2644	1.4406	0.1497
SDV = TT ver [i] : Tense = stem+ing	0.7395	0.2741	2.6979	0.0070
SDV = TB ver [i] (Tense = stem)	11.7611	0.2780	42.2991	< 0.0001
SDV = TB ver [i] : Tense = stem+s	−0.9430	0.2233	−4.2235	< 0.0001
SDV = TB ver [i] : Tense = stem+d	−0.5270	0.2583	−2.0400	0.0413
SDV = TB ver [i] : Tense = stem+ing	−0.6493	0.2662	−2.4390	0.0147
SDV = TT hor [ɑ] (Tense = stem)	−2.3130	0.2121	−10.9076	< 0.0001
SDV = TT hor [ɑ] : Tense = stem+s	0.1184	0.2695	0.4392	0.6605
SDV = TT hor [ɑ] : Tense = stem+d	0.2741	0.3130	0.8760	0.3811
SDV = TT hor [ɑ] : Tense = stem+ing	0.4097	0.3274	1.2511	0.2109
SDV = TT ver [ɑ] (Tense = stem)	3.6150	0.2327	15.5326	< 0.0001
SDV = TT ver [ɑ] : Tense = stem+s	−3.3122	0.2984	−11.0987	< 0.0001
SDV = TT ver [ɑ] : Tense = stem+d	−3.0447	0.3462	−8.7956	< 0.0001
SDV = TT ver [ɑ] : Tense = stem+ing	0.4263	0.3562	1.1966	0.2315
SDV = TB ver [ɑ] (Tense = stem)	5.2527	0.2260	23.2376	< 0.0001
SDV = TB ver [ɑ] : Tense = stem+s	−1.8583	0.2829	−6.5697	< 0.0001
SDV = TB ver [ɑ] : Tense = stem+d	−1.0930	0.3333	−3.2797	0.0010
SDV = TB ver [ɑ] : Tense = stem+ing	0.0967	0.3501	0.2761	0.7825

**Table 4:** Summary of smooth terms for the model fitted to tongue sensor movements in the midsaggital plane during the production of [i]. The lexical predictor was **paradigmatic probability**. TT = tongue tip, TB = tongue body, ver = vertical movements, hor = horizontal movements. Effective degrees of freedom (edf) substantially greater than 1 indicate a non-linear relationship between smooth and dependent variable. P-values smaller than 0.0001 are regarded to support significant effects

smooth terms	edf	ref.df	F-value	p-value
s(time): TT hor [i]	2.4192	2.7640	108.9699	< 0.0001
s(time): TT ver [i]	2.8749	2.9878	220.3148	< 0.0001
s(time): TB hor [i]	2.8171	2.9742	210.4846	< 0.0001
s(time): TB ver [i]	2.9284	2.9960	283.4926	< 0.0001
s(time): TT hor [ɑ]	2.9147	2.9943	480.2161	< 0.0001
s(time): TT ver [ɑ]	2.9371	2.9969	583.1954	< 0.0001
s(time): TB hor [ɑ]	2.7648	2.9576	271.6577	< 0.0001
s(time): TB ver [ɑ]	2.8765	2.9880	338.2444	< 0.0001
s(paradigmatic probability): TT hor [i]	1.6384	1.8681	10.8501	0.0021
s(paradigmatic probability): TT ver [i]	1.9775	1.9994	45.6623	< 0.0001
s(paradigmatic probability): TB hor [i]	1.4258	1.6689	6.5057	0.0152
s(paradigmatic probability): TB ver [i]	1.9420	1.9962	20.4445	< 0.0001
s(paradigmatic probability): TT hor [ɑ]	1.5162	1.7654	1.9412	0.2224
s(paradigmatic probability): TT ver [ɑ]	1.9771	1.9994	107.8204	< 0.0001
s(paradigmatic probability): TB hor [ɑ]	1.9035	1.9905	23.0086	< 0.0001
s(paradigmatic probability): TB ver [ɑ]	1.9712	1.9991	48.5218	< 0.0001
s(participant)	23.9046	24.0000	12306.7123	< 0.0001
s(base)	53.1517	54.0000	7304.1250	< 0.0001

**Fig. 2:**
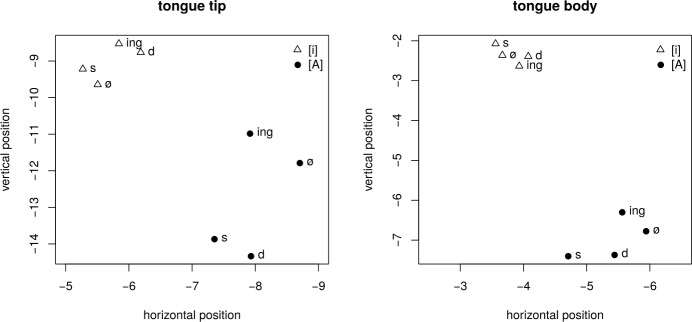
Position of the tongue tip (**left**) and tongue body (**right**) sensor at the onset of the vowel, for [i] (triangles) and [ɑ] (disks) by morphological condition (ø: stem, s: third person singular, d: past tense, ing: gerund). Axis are in millimeters. The relative position between [i] and [ɑ] has been reduced to increase the discriminability of the tense effects. The opening of the oral cavity is to the left. Random effects were excluded for prediction

Interpretation of the large number of coefficients in Table 3 is facilitated by visualization in Fig. 2. The left panel of Fig. 2 presents the predicted positions at the onset of the vowel (the intercepts at time zero) of the tongue tip sensor, the right panel the intercepts for the tongue body sensor. Triangles represent intercepts for [i], filled circles intercepts for [ɑ]. Individual points represent the relative onset positions of the tongue tip and tongue body as affected by tense. The relative position between [i] and [ɑ] has been reduced to increase the discriminability between the points representing the effect of tense.^2^

**Fig. 3:**
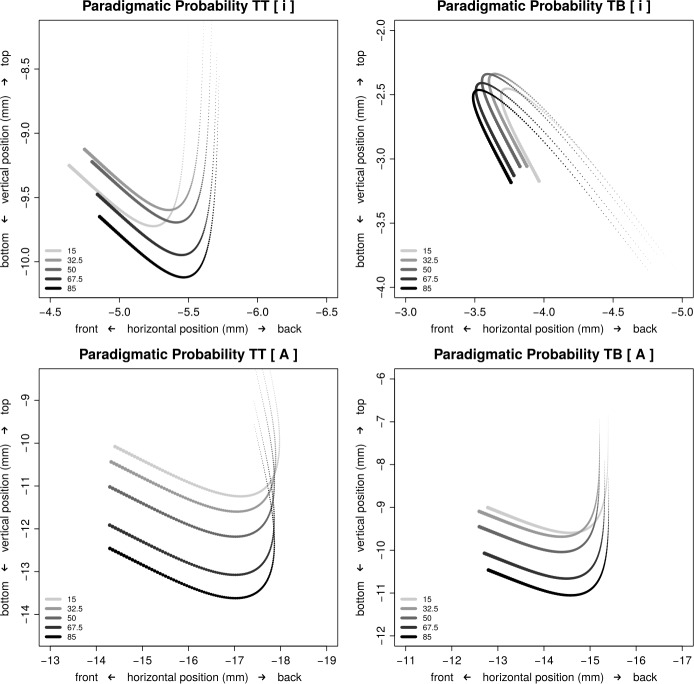
Articulatory trajectories in the midsaggital plane of the tongue tip sensor (**left panels**) and the tongue body sensor (**right panels**) sensors during the articulation of the vowels [i] (**top row**) and [ɑ] (**bottom row**). The x-axis represents the distance to the horizontal reference point near the lips, the y-axis represents the distance to the palate. The opening of the oral cavity is to the left. Line width represents time, with greater line width indicating earlier points in time. The 5 curves in a given panel represent quantiles of paradigmatic probability, here, darker shades of gray represent larger quantiles. Random effects were excluded for prediction

Table 4 presents the summary of the smooth terms of the model. The last block of this table concerns the by-subject and by-base random intercepts, both are well supported. As they are not of theoretical interest, we will not discuss these random effects further. The first block of smooths in Table 4 specifies how position varies over time as a function of SDV (the factor defining the eight combinations of sensor, dimension, and vowel). These smooths lay down the foundation for the trajectories in the midsaggital plane shown in Fig. 3. The precise shape of these trajectories varies with paradigmatic probability. The second block of Table 4 evaluates the effect of paradigmatic probability for the 8 levels of SDV. In 5 out of 8 cases, there is good reason to assume that indeed paradigmatic probability is co-determining articulation. The trajectories shown in Fig. 3 present the joint effect of the smooths for time and paradigmatic probability. Smooths are shown for each of the four vowel and sensor combinations, with horizontal position on the X-axis and vertical position on the Y-axis. Time is indicated by means of line width, with greater line width indicating earlier points in time. The modulating effect of paradigmatic probability is shown by graphing the curve at five percentiles: the 15th, 32.5th, 50th, 67.5th, and 85th percentile of paradigmatic probability. Darker shades of gray represent higher values of paradigmatic probability.

First consider the upper panels of Fig. 3, which display the articulatory trajectories for the [i] vowel. The trajectories for the tongue tip sensor (left) are U-shaped, whereas those for the tongue body sensor are inverse U-shaped. This suggests that we are observing anti-phasic coupled motion during [i]: when the tongue tip moves down, at the same time, the tongue body moves up. In other words, the movement pattern registered here is truly tied to the tongue, and not to jaw movement.

During the articulation of the [i] vowel, the tongue tip moves further down into the mouth as paradigmatic probability increases. Furthermore, the tongue tip is slightly retracted, whereas the tongue body sensor is fronted slightly (an effect that yielded a relatively high p = 0.0152). This raises the question of how to understand this frequency effect. We first observe that producing an [i] vowel requires finding a good balance between staying away of the [j] semi-vowel, which requires the tongue to move closer to the palate compared to [i]. In spite of the durational cue for [i], realizing the [i] still requires good resonance while staying away from the lower articulatory positions which give rise to the [ɪ] vowel. Given this balancing act, there are two ways in which the lowering effect of paradigmatic probability can be understood. The first interpretation is consistent with the *Smooth Signal Reduction Hypothesis*: with increasing (paradigmatic) probability, the [i] vowel is realized with more centralization. The second interpretation is consistent with the *Paradigmatic Enhancement Hypothesis* (and the practice hypothesis): words with small paradigmatic probability are realized with too narrow a vocal tract, resulting in too little resonance; as experience with articulating the word increases, [i] vowels are realized with less constriction and more vocalic resonance. The first interpretation zooms in on movement towards the schwa, the second interpretation focuses on movement away from a possible constriction. The second interpretation has perhaps slightly better credentials: paradigmatic probability outperforms whole-word frequency as a predictor of articulation, and paradigmatic effects invariably lead to strengthening of the signal and not weakening. If the first interpretation is correct, then the prediction follows that for the low [ɑ] vowel, we should observe raising towards the schwa.

The lower panels of Fig. 3 show that this prediction of the *Smooth Signal Redundancy Hypothesis* for the articulation of the low vowel [ɑ] is incorrect. The most parsimonious interpretation of the effect of paradigmatic probability therefore is that what we are witnessing is the improvement of motor skills with practice. It is also noteworthy that the tongue tip and tongue body sensors are not showing anti-phasic movements. This suggests that it is not so much the tongue shape that is being modulated during articulation, but rather that we are observing the effect of the lowering and raising of the jaw.

## General discussion

In this study, we reviewed theories of word structure in linguistics and models of lexical processing in psychology. In linguistics, theories of word structure attempt to offer a parsimonious account of the word structures that are possible in a given language.

Most researchers currently adopt some form of realizational morphology, which works with sets of inflectional and derivational features that have to be expressed in combinations of stems and exponents. In this approach, there is no need to list regular complex words, as their forms can be produced on the fly from rules and/or inheritance hierarchies.

Many computational models for word production in psychology have adopted some form of realizational morphology. In addition they have adopted the postulate that because rules can assemble complex words, complex words do not have representations of their own in lexical memory. Whereas frequency effects observed for complex words across many comprehension studies (see, e.g., Baayen et al. 1997; Giraudo and Orihuela 2015; Schmidtke et al. 2017) have been interpreted as evidence for complex words having their own representation in lexical memory, the evidence for frequency effects in chronometric studies is not as clear (see, e.g. Levelt et al. 1999; Janssen et al. 2008). However, studies of the acoustic signal, i.e., of the speech produced, and of the articulatory process itself, provide evidence that complex words are more than simple combinations of parts (see, e.g., Baayen et al. 2019; Tomaschek et al. 2019, 2018c). The subtle meanings of complex words, including their pragmatic and social functions, have far-reaching consequences for phonetic detail (Hawkins 2003; Drager 2011; Podlubny et al. 2015). To our knowledge, none of the realizational computational models that have been proposed in the literature have been able to properly predict these findings.

The present study adds to the growing body of research on the phonetics of complex words by addressing the question of whether the frequency with which regular inflected words are used co-determines how they are articulated. Two complementary theories make opposite predictions. One theory, under the banner of the *Smooth Signal Redundancy Hypothesis* (Aylett and Turk 2004) and the *Uniform Information Density Hypothesis* (Frank and Jaeger 2008; Jaeger 2010), predicts that inflected words with higher syntagmatic probability should be produced with reduced articulatory trajectories, as in context higher frequency words are informationally more redundant. The other theory, under the banner of the *Paradigmatic Enhancement Hypothesis*, predicts that words with higher paradigmatic probability should be produced with enhanced articulatory trajectories, the idea here being that words that are more probable within a paradigm can be articulated with more confidence (Kuperman et al. 2007). Since such words have had more extensive motor practice, the articulatory gestures for these words can be executed with enhanced kinematic skills (Tomaschek et al. 2018c). Enhanced kinematic skills result from greater practice and allow speakers to achieve more extreme articulatory positions, in addition to smoother gestural transitions (Tiede et al. 2011), faster articulatory velocity (Tomaschek et al. 2018a) and greater articulatory precision (Tomaschek et al. 2020).

Using electromagnetic articulography to study articulatory trajectories in the midsaggital plain for English regular inflected verbs, the present study was able to document clear frequency effects. For verbs with [i] as stem vowel, the tongue tip moves down more and further away from the palate as the paradigmatic probability of the word form increases. For verbs with [ɑ] as stem vowel, the jaw moves further down for words with higher paradigmatic probability. The enhanced articulation for the [ɑ] straightforwardly contradicts the *Smooth Signal Redundancy Hypothesis*. The interpretation of the lowering of the tongue for verbs with [i] as stem vowel can be construed as supporting the *Smooth Signal Redundancy Hypothesis*, as it could signal a form of reduction towards [ə]. However, it can also be seen as improved motor control that avoids a constriction and results in a resonant [i]. This second interpretation is supported by the finding that paradigmatic probability is a better predictor than word frequency, in combination with the finding that it is paradigmatic measures that give rise to strengthening and not syntagmatic probabilities or isolated word frequencies (see, e.g., Lõo et al. 2018; Bell et al. 2019; Cohen 2014b; Kuperman et al. 2007; Tucker et al. 2019; Tomaschek et al. 2019; Cohen 2014a, 2015; Sims 2016). The interpretation of the lowering of the [i] as reflecting articulatory optimization also dovetails well with the optimization visible for the [ɑ] vowel. Importantly, effects of enhancement of acoustic durations observed in speech corpora (Tucker et al. 2019; Tomaschek et al. 2019) suggest that the present effects of enhancement are not restricted to the laboratory setting.

The present results point to a gap in our current understanding of the relationship between probability and performance in speech. Whereas it seems that syntagmatic probability leads to shortening and reduction, it also seems that paradigmatic probability gives rise to lengthening and enhancement. How are these seemingly obvious findings to be reconciled? Is there an underlying principle that can unify the opposite predictions of the *Smooth Signal Redundancy Hypothesis* and the *Paradigmatic Enhancement Hypothesis*?

Shortening and reduction of higher frequency words has been explained in various ways. According to Bell et al. (2009), Gahl (2008), Buz and Jaeger (2016), higher frequency words are more readily available in the mental lexicon, allowing for faster timing of cognitive preparation processes, which in turn are assumed to give rise to shorter acoustic durations and more reduction. The number of phonological neighbors has also been found to be an important predictor for the modulation of phonetic detail; most studies report enhancement effects in relation to phonological neighborhood density; i.e. words are articulated longer and with more peripheral vowels when there is larger competition with phonologically similar words (Wright 2004; Munson 2001; Scarborough 2003; Buz and Jaeger 2016; Fricke et al. 2016) (for a contradictory finding see (Gahl et al. 2012)). Minimization of effort (Zipf 1949; Lindblom 1990) and smoothing informativity of the signal in time (Aylett and Turk 2004, 2006) provide further interesting perspectives on the negative correlation of frequency with duration and signal richness.

However, there is yet another perspective that can be added to this list. Higher frequency words tend to have more meanings (Köhler 1986), they appear in more diverse contexts (Adelman et al. 2006; Linke and Ramscar 2020), and they are found in more word n-grams.^3^ From a *Discriminative Learning* perspective (Ramscar et al. 2010, 2013b; Ramscar and Yarlett 2007), the words with which a given word co-occurs in word n-grams constitute cues that compete during learning. Since higher frequency words occur with more (contextual) cues, higher-frequency words are potentially less learnable than lower-frequency words. Furthermore, it is more likely that for higher-frequency words multiple meanings are competing for expression in the same form (see Chuang et al. 2020, for the frailty induced by homophones in multilingual learning). Whereas their rich syntagmatic diversity has adverse consequences for the learning of higher-frequency words, a higher paradigmatic probability is an indicator of reduced competition from paradigmatic competitors, and hence of increased learnability.

From this learning perspective, the *Smooth Signal Redundancy Hypothesis* and the *Paradigmatic Enhancement Hypothesis* are describing exactly the same phenomenon, only from different points of view. Higher word frequency implies greater competition of contextual cues, but a higher paradigmatic probability implies reduced competition from paradigmatic competitors. The unifying theme is that greater learnability is associated with longer durations and more skillful articulation, whereas reduced learnability comes with decreasing acoustic durations and more centralized and less effortful realizations. Thus, when learnability tends towards zero, the rule holds that the unlearnable cannot have any acoustic realization: “Wovon man nicht sprechen kann, darüber muss man schweigen”^4^ (Wittgenstein 1922). Which leaves us with the question and an area for future research, how do we best quantify learnability and apply it to our models of speech production?

From our perspective, proposals such as *Discriminative Learning* (Ramscar et al. 2010) and the *Discriminative Lexicon* model (Baayen et al. 2018b) are interesting and fruitful attempts in the right direction, as they do not work with lexical representations for form and meaning that are stored in some list-like dictionary. Rather, the networks are the linguistic memory. A word’s meaning is generated on the fly from visual or acoustic input, and a word’s form is generated on the fly given the message the speaker is seeking to encode. Baayen et al. (2018b) and Chuang et al. (2020) show that algorithmically, it is possible to understand and produce morphologically complex words without requiring theoretical constructs such as stems, affixes, and exponents.

We conclude with the observation that the robust frequency effects observed in the articulation of complex words caution against projecting parsimony in linguistic analysis onto the mental lexicon. The present findings are congruent with *construction morphology* (Booij 2010), the latest developments in *Word and Paradigm Morphology* (Blevins et al. 2015; Blevins 2016) which heavily rely on discriminative approaches, and with usage-based approaches to language and language processing (see e.g. Bybee 2010), albeit they challenge the implicit assumptions regarding compositionality embodied in many of the latter approaches (see Ramscar and Port 2016, for a critical review of compositional approaches). An important challenge for both linguistic morphology and cognitive modeling is to further develop our theories so that they generate precise and falsifiable quantitative predictions for articulatory trajectories, along the work by Hickok (2012, 2014). We anticipate that in order to achieve this goal, it will be necessary to investigate how lexical, inflectional, derivational, and pragmatic meanings are realized in the phonetics of complex words, without intervening discrete units set up in such a way that they render form and meaning invisible to each other.

## Figures and Tables

**Fig. 4:**
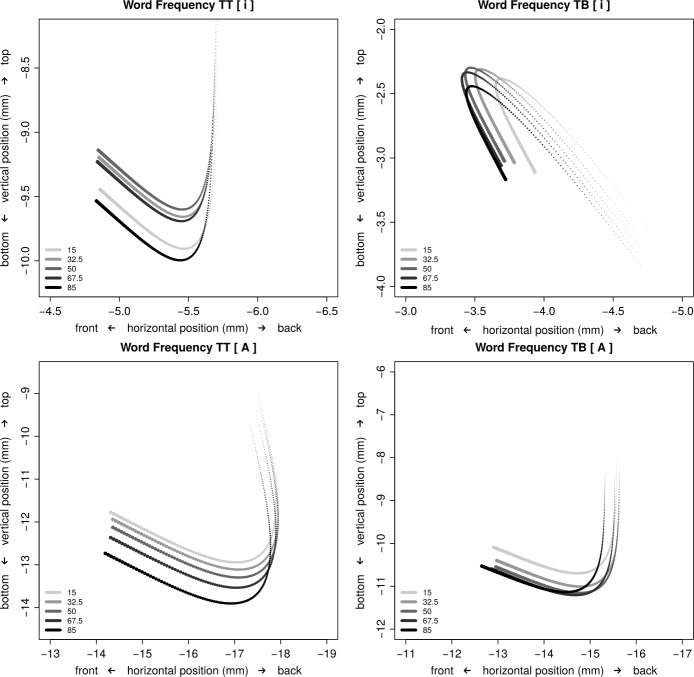
Articulatory trajectories in the midsaggital plane of two tongue sensors during the articulation of the stem vowel [i] (top row) and [ɑ] (bottom row) of English verbs. The left column visualizes these trajectories for the tongue tip sensor, the right column for the tongue body movements. The x-axis represents the distance to the horizontal reference point, the y-axis represents the distance to the palate. The opening of the oral cavity is to the left. The thickness of the trajectories represents position across time, with the thick end representing the onset of the vowel and the thin end representing the offset of the vowel. Shading represents the deciles of **whole-word frequency**, with black = 0.85 decile and light grey = 0.15 decile. Random effects were excluded for prediction

## Appendix: Summaries and plots for whole-word frequency

Figure 4 illustrates the effect of whole-word frequency on articulatory trajectories. The summary of parametric coefficient is presented in Table 5. Table 6 summarizes the non-linear effects.

**Table 5:** Summary of parametric coefficients for the model fitted to tongue sensor movements in the midsaggital plane. The lexical predictor was **whole-word frequency**. SDV = Sensor by Dimension by Vowel, TT = tongue tip, TB = tongue body, ver = vertical movements, hor = horizontal movements

parametric coefficients	estimate	std. error	t-value	p-value
(Intercept)	−14.7510	0.2899	−50.8754	< 0.0001
TB hor [i]	10.8316	0.2569	42.1635	< 0.0001
TB ver [ɑ]	4.6915	0.1565	29.9768	< 0.0001
TB ver [i]	11.8961	0.2555	46.5669	< 0.0001
TT hor [ɑ]	−2.1626	0.1531	−14.1206	< 0.0001
TT hor [i]	9.3206	0.2563	36.3727	< 0.0001
TT ver [ɑ]	2.7278	0.1622	16.8184	< 0.0001
TT ver [i]	5.3013	0.2574	20.5994	< 0.0001
Tense = stem+d	0.6210	0.1804	3.4427	0.0006
Tense = stem+s	1.2803	0.1583	8.0893	< 0.0001
Tense = stem+ing	0.4334	0.1861	2.3295	0.0198
SDV = TB hor [i] : Tense = stem+d	−1.0616	0.1998	−5.3134	< 0.0001
SDV = TB ver [ɑ] : Tense = stem+d	−0.1744	0.2298	−0.7590	0.4479
SDV = TB ver [i] : Tense = stem+d	−0.6762	0.1948	−3.4710	0.0005
SDV = TT hor [ɑ] : Tense = stem+d	0.0962	0.2252	0.4269	0.6694
SDV = TT hor [i] : Tense = stem+d	−1.1716	0.1976	−5.9292	< 0.0001
SDV = TT ver [ɑ] : Tense = stem+d	−1.6940	0.2430	−6.9721	< 0.0001
SDV = TT ver [i] : Tense = stem+d	0.3798	0.2019	1.8810	0.0600
SDV = TB hor [i] : Tense = stem+s	−1.2253	0.1752	−6.9938	< 0.0001
SDV = TB ver [ɑ] : Tense = stem+s	−1.0828	0.2001	−5.4103	< 0.0001
SDV = TB ver [i] : Tense = stem+s	−0.9958	0.1712	−5.8162	< 0.0001
SDV = TT hor [ɑ] : Tense = stem+s	−0.0176	0.2004	−0.0877	0.9301
SDV = TT hor [i] : Tense = stem+s	−0.9756	0.1735	−5.6243	< 0.0001
SDV = TT ver [ɑ] : Tense = stem+s	−2.1082	0.2126	−9.9184	< 0.0001
SDV = TT ver [i] : Tense = stem+s	−0.6590	0.1783	−3.6951	0.0002
SDV = TB hor [i] : Tense = stem+ing	−0.8314	0.2045	−4.0656	< 0.0001
SDV = TB ver [ɑ] : Tense = stem+ing	1.1117	0.2390	4.6519	< 0.0001
SDV = TB ver [i] : Tense = stem+ing	−0.8253	0.1997	−4.1322	< 0.0001
SDV = TT hor [ɑ] : Tense = stem+ing	0.2608	0.2351	1.1090	0.2674
SDV = TT hor [i] : Tense = stem+ing	−0.6632	0.2026	−3.2738	0.0011
SDV = TT ver [ɑ] : Tense = stem+ing	1.9943	0.2407	8.2869	< 0.0001
SDV = TT ver [i] : Tense = stem+ing	0.5748	0.2098	2.7391	0.0062

**Table 6:** Summary of smooth terms for the model fitted to tongue sensor movements in the midsaggital plane during the production of [i]. The lexical predictor was **whole-word frequency**. TT = tongue tip, TB = tongue body, ver = vertical movements, hor = horizontal movements. Effective degrees of freedom (edf) substantially greater than 1 indicate a non-linear relationship between smooth and dependent variable

smooth terms	edf	ref.df	F-value	p-value
s(time): TB hor [ɑ]	2.7721	2.9602	281.3910	< 0.0001
s(time): TB hor [i]	2.8188	2.9746	207.3173	< 0.0001
s(time): TB ver [ɑ]	2.8805	2.9888	347.3624	< 0.0001
s(time): TB ver [i]	2.9278	2.9959	281.6231	< 0.0001
s(time): TT hor [ɑ]	2.9160	2.9945	485.9599	< 0.0001
s(time): TT hor [i]	2.4159	2.7617	108.0798	< 0.0001
s(time): TT ver [ɑ]	2.9387	2.9970	611.4520	< 0.0001
s(time): TT ver [i]	2.8749	2.9878	217.0389	< 0.0001
s(word frequency): TB hor [ɑ]	1.8829	1.9850	6.9371	0.0261
s(word frequency): TB hor [i]	1.9379	1.9958	18.6328	0.0001
s(word frequency): TB ver [ɑ]	1.9361	1.9949	36.5984	< 0.0001
s(word frequency): TB ver [i]	1.9468	1.9968	19.0345	0.0001
s(word frequency): TT hor [ɑ]	1.7604	1.9405	2.9305	0.2095
s(word frequency): TT hor [i]	1.0011	1.0022	0.0986	0.7544
s(word frequency): TT ver [ɑ]	1.7777	1.9489	23.4363	< 0.0001
s(word frequency): TT ver [i]	1.9902	1.9998	105.0520	< 0.0001
s(Participant)	23.9066	24.0000	13106.4578	< 0.0001
s(Base)	53.0458	54.0000	7971.4828	< 0.0001

## Footnotes

1 To avoid the problem of the quotient rule for logarithms, we used ranked predictors. For model comparisons, we refitted the model with ranked paradigmatic probability.

2 We observe shifts in the intercept of the sensor positions between the different conditions and the stem condition (represented by “ø”). Anticipating [ɱ] and [d], the tongue tip is raised and retracted during [i] production. Anticipating [s], the tongue is shifted to the front and raised. The same effect can be observed when the tongue body anticipates [s] during [i] production. While the tongue body is also retracted, when anticipating [ɱ] and [d], it is a little bit lowered before [ɱ]. During [ɑ] production, the tongue tip is systematically fronted anticipating all exponents. It is lower anticipating [s] and [d] and higher anticipating [ɱ]. The same pattern can be observed for the tongue body, even though the differences between the stem condition and the suffixed condition are smaller than in the tongue tip. One potential reason why we observe lower positions of the tongue before [s] and [d] is that these consonants were articulated with the tongue blade rather than the tongue tip. When these consonants are articulated by the tongue blade, then the tongue tip gets fronted and lowered due to the raising of the blade.

3 For instance, the correlation between Celex word form unigram frequency and the number of 4-grams in the google-1T n-gram frequency lists in which the word forms occur is 0.66 (*p*<0.0001).

4 Whereof one cannot speak, thereof one must be silent.

## References

[CR1] Adelman J., Brown G., Quesada J. (2006). Contextual diversity, not word frequency, determines word-naming and lexical decision times. Psychological Science.

[CR2] Aylett M., Turk A. (2004). The smooth signal redundancy hypothesis: A functional explanation for relationships between redundancy, prosodic prominence, and duration in spontaneous speech. Language and Speech.

[CR3] Aylett M., Turk A. (2006). Language redundancy predicts syllabic duration and the spectral characteristics of vocalic syllable nuclei. Journal of the Acoustical Society of America.

[CR4] Baayen R. H., Dijkstra T., Schreuder R. (1997). Singulars and plurals in Dutch: Evidence for a parallel dual-route model. Journal of Memory and Language.

[CR5] Baayen R. H., McQueen J., Dijkstra T., Schreuder R., Baayen R. H., Schreuder R. (2003). Frequency effects in regular inflectional morphology: Revisiting Dutch plurals. Morphological structure in language processing.

[CR6] Baayen R. H., Levelt W. M., Schreuder R., Ernestus M. (2008). Paradigmatic structure in speech production. Proceedings Chicago Linguistics Society 43.

[CR7] Baayen R. H., Wurm L. H., Aycock J. (2008). Lexical dynamics for low-frequency complex words. A regression study across tasks and modalities. The Mental Lexicon.

[CR8] Baayen R. H., Milin P., Filipović Ðurdjević D., Hendrix P., Marelli M. (2011). An amorphous model for morphological processing in visual comprehension based on naive discriminative learning. Psychological Review.

[CR9] Baayen R. H., Vasishth S., Bates D., Kliegl R. (2017). The cave of shadows. Addressing the human factor with generalized additive mixed models. Journal of Memory and Language.

[CR10] Baayen H., Rij J., De Cat C., Wood S., Speelman D., Heylen K., Geeraerts D. (2018). Autocorrelated errors in experimental data in the language sciences: Some solutions offered by generalized additive mixed models. Mixed-effects regression models in linguistics.

[CR11] Baayen R. H., Chuang Y.-Y., Blevins J. P. (2018). Inflectional morphology with linear mappings. The Mental Lexicon.

[CR12] Baayen, R. H., Chuang, Y.-Y., Shafaei-Bajestan, E., & Blevins, J. (2019). The discriminative lexicon: A unified computational model for the lexicon and lexical processing in comprehension and production grounded not in (de)composition but in linear discriminative learning. *Complexity*. 10.1155/2019/4895891.

[CR13] Bard E. G., Anderson A. H., Sotillo C., Aylett M., Doherty-Sneddon G., Newlands A. (2000). Controlling the intelligibility of referring expressions in dialogue. Journal of Memory and Language.

[CR14] Bell A., Brenier J. M., Gregory M., Girand C., Jurafsky D. (2009). Predictability effects on durations of content and function words in conversational English. Journal of Memory and Language.

[CR15] Bell, M. J., Ben Hedia, S., & Plag, I. (2019). How morphological structure affects phonetic realization in English compound nouns. *Morphology*, 1–34.

[CR16] Bien H., Levelt W. J., Baayen R. H. (2005). Frequency effects in compound production. Proceedings of the National Academy of Sciences.

[CR17] Bien H., Baayen R. H., Levelt W. J. (2011). Frequency effects in the production of Dutch deverbal adjectives and inflected verbs. Language and Cognitive Processes.

[CR18] Blevins J. P. (2016). Word and paradigm morphology.

[CR19] Blevins J. P., Ackerman F., Malouf R., Ramscar M., Harley H., Siddiqi D. (2015). Morphology as an adaptive discriminative system. Morphological metatheory.

[CR20] Bonami O., Stump G. T., Hippisley A., Stump G. T. (2016). Paradigm function morphology. The Cambridge Handbook of Morphology.

[CR21] Booij G. (2010). Construction morphology. Language and Linguistics Compass.

[CR22] Brandt E., Andreeva B., Möbius B., Calhoun S., Escudero P., Tabain M., Warren P. (2019). Information density and vowel dispersion in the productions of Bulgarian L2 speakers of German. Proceedings of the 19th International Congress of Phonetic Sciences.

[CR23] Browman C., Goldstein L. (1986). Towards an articulatory phonology. Phonology.

[CR24] Buz E., Jaeger T. F. (2016). The (in)dependence of articulation and lexical planning during isolated word production. Language, Cognition and Neuroscience.

[CR25] Bybee J. (2010). Language, usage and cognition.

[CR26] Cho T. (2001). Effects of morpheme boundaries on intergestural timing: Evidence from Korean. Phonetica.

[CR27] Cholin J., Schiller N. O., Levelt W. J. M. (2004). The preparation of syllables in speech production. Journal of Memory and Language.

[CR28] Cholin J., Levelt W. J., Schiller N. O. (2006). Effects of syllable frequency in speech production. Cognition.

[CR29] Chuang Y.-Y., Lõo K., Blevins J. P., Baayen R., Körtvélyessy L., Štekauer P. (2020). Estonian case inflection made simple. A case study in Word and Paradigm morphology with Linear Discriminative Learning. Complex Words.

[CR30] Chuang Y.-Y., Bell M. J., Baayen R. (2020). Bilingual and multilingual mental lexicon: a modeling study with Linear Discriminative Learning. Language Learning.

[CR31] Cohen, C. (2014a). *Combining structure and usage patterns in morpheme production: Probabilistic effects of sentence context and inflectional paradigms*. PhD dissertation, University of California, Berkeley.

[CR32] Cohen C. (2014). Probabilistic reduction and probabilistic enhancement. Morphology.

[CR33] Cohen C. (2015). Context and paradigms: two patterns of probabilistic pronunciation variation in Russian agreement suffixes. Mental Lexicon.

[CR34] Cohen Priva U. (2015). Informativity affects consonant duration and deletion rates. Laboratory Phonology.

[CR35] Dell G. S. (1986). A spreading-activation theory of retrieval in sentence production. Psychological Review.

[CR36] Dell G. S. (1990). Effects of frequency and vocabulary type on phonological speech errors. Language and Cognitive Processes.

[CR37] Dell G. S., Schwartz M. F., Martin N., Saffran E. M., Gagnon D. A. (1997). Lexical access in aphasic and nonaphasic speakers. Psychological Review.

[CR38] Dell G. S., Martin N., Schwartz M. F. (2007). A case-series test of the interactive two-step model of lexical access: Predicting word repetition from picture naming. Journal of Memory and Language.

[CR39] Drager K. K. (2011). Sociophonetic variation and the lemma. Journal of Phonetics.

[CR40] Ernestus, M. (2000). *Voice assimilation and segment reduction in casual Dutch – A corpus-based study of the phonology-phonetics interface*. Doctoral dissertation, Vrije Unversiteit te Amsterdam.

[CR41] Ernestus M., Baayen R. H., Schreuder R. (2002). The recognition of reduced word forms. Brain and Language.

[CR42] Fasiolo M., Wood S. N., Zaffran M., Nedellec R., Goude Y. (2020). Fast calibrated additive quantile regression. Journal of the American Statistical Association.

[CR43] Foygel D., Dell G. S. (2000). Models of impaired lexical access in speech production. Journal of Memory and Language.

[CR44] Frank A. F., Jaeger T. F. (2008). Speaking rationally: Uniform information density as an optimal strategy for language production. Proceedings of the annual meeting of the cognitive science society.

[CR45] Frauenfelder U. H., Schreuder R., Booij G. E., Marle J. v. (1992). Constraining psycholinguistic models of morphological processing and representation: the role of productivity. Yearbook of morphology 1991.

[CR46] Fricke M., Baese-Berk M. M., Goldrick M. (2016). Dimensions of similarity in the mental lexicon. Language, Cognition and Neuroscience.

[CR47] Friedman L., Wall M. (2005). Graphical views of suppression and multicollinearity in multiple regression. The American Statistician.

[CR48] Gafos A., Hoole P., Roon K., Zeroual C. (2010). Variation in overlap and phonological grammar in Moroccan Arabic clusters. Laboratory Phonology.

[CR49] Gahl S. (2008). Thyme and Time are not homophones. Word durations in spontaneous speech. Language.

[CR50] Gahl S., Yao Y., Johnson K. (2012). Why reduce? Phonological neighborhood density and phonetic reduction in spontaneous speech. Journal of Memory and Language.

[CR51] Giraudo H., Orihuela K., Pirrelli V., Marzi C., Ferro M. (2015). Visual word recognition of morphologically complex words: effects of prime word and root frequency. Proceedings of the NetWordS final conference ‘NetWordS’.

[CR52] Guenther F. H. (1995). Speech sound acquisition, coarticulation, and rate effects in a neural network model of speech production. Biological Cybernetics.

[CR53] Hall K. C., Hume E., Jaeger T. F., Wedel A. (2018). The role of predictability in shaping phonological patterns. Linguistics Vanguard.

[CR54] Halle M., Marantz A. (1994). Some key features of distributed morphology. MIT Working Papers in Linguistics.

[CR55] Hanique I., Ernestus M. (2012). The role of morphology in acoustic reduction. Lingue E Linguaggio.

[CR56] Hastie T., Tibshirani R. (1990). Generalized additive models.

[CR57] Hawkins S. (2003). Roles and representations of systematic fine phonetic detail in speech understanding. Journal of Phonetics.

[CR58] Hickok G. (2012). Computational neuroanatomy of speech production. Nature Reviews Neuroscience.

[CR59] Hickok G. (2014). The architecture of speech production and the role of the phoneme in speech processing. Language, Cognition and Neuroscience.

[CR60] Hockett C. (1954). Two models of grammatical description. Word.

[CR61] Jackendoff R. (1975). Morphological and semantic regularities in the lexicon. Language.

[CR62] Jaeger T. F. (2010). Redundancy and reduction: Speakers manage syntactic information density. Cognitive Psychology.

[CR63] Janssen N., Bi Y., Caramazza A. (2008). A tale of two frequencies: Determining the speed of lexical access for Mandarin Chinese and English compounds. Language and Cognitive Processes.

[CR64] Jescheniak J. D., Levelt W. J. (1994). Word frequency effects in speech production: Retrieval of syntactic information and of phonological form. Journal of Experimental Psychology: Learning, Memory, and Cognition.

[CR65] Johnson K. (2004). Massive reduction in conversational American English. Spontaneous speech: data and analysis. Proceedings of the 1st session of the 10th international symposium.

[CR66] Juola P. (1998). Measuring linguistic complexity: The morphological tier. Journal of Quantitative Linguistics.

[CR67] Kemps R., Ernestus M., Schreuder R., Baayen H. (2004). Processing reduced word forms: the suffix restoration effect. Brain and Language.

[CR68] Keune K., Ernestus M., Hout R. V., Baayen R. H. (2005). Variation in Dutch: From written MOGELIJK to spoken MOK. Corpus Linguistics and Linguistic Theory.

[CR69] Kittredge A. K., Dell G. S., Verkuilen J., Schwartz M. F. (2008). Where is the effect of frequency in word production? Insights from aphasic picture-naming errors. Cognitive Neuropsychology.

[CR70] Koenker R. (2005). Quantile regression.

[CR71] Köhler R. (1986). Zur linguistischen Synergetik: Struktur und Dynamik der Lexik.

[CR72] Kuperman V., Pluymaekers M., Ernestus M., Baayen H. (2007). Morphological predictability and acoustic duration of interfixes in Dutch compounds. The Journal of the Acoustical Society of America.

[CR73] Le Maguer S., Möbius B., Steiner I. (2016). Toward the use of information density based descriptive features in HMM based speech synthesis. Proceedings of the 8th International Conference on Speech Prosody.

[CR74] Lee-Kim S.-I., Davidson L., Hwang S. (2012). Morphological effects on the darkness of English intervocalic /l/. Laboratory Phonologya.

[CR75] Levelt W. J. M., Schriefers H., Vorberg D., Meyer A. S., Pechmann T., Havinga J. (1991). The time course of lexical access in speech production: A study of picture naming. Psychological Review.

[CR76] Levelt W. J., Roelofs A., Meyer A. S. (1999). A theory of lexical access in speech production. The Behavioral and Brain Sciences.

[CR77] Lindblom B., Marchal A., Hardcastle W. (1990). Explaining phonetic variation: A sketch of the H&H theory. Speech production and speech modelling.

[CR78] Linke M., Ramscar M. (2020). How the probabilistic structure of grammatical context shapes speech. Entropy.

[CR79] Lohmann A. (2018). Cut (n) and cut (v) are not homophones: Lemma frequency affects the duration of noun–verb conversion pairs. Journal of Linguistics.

[CR80] Lõo K., Järvikivi J., Tomaschek F., Tucker B. V., Baayen R. H. (2018). Production of Estonian case-inflected nouns shows whole-word frequency and paradigmatic effects. Morphology.

[CR81] Malisz, Z., Brandt, E., Möbius, B., Oh, Y. M., & Andreeva, B. (2018). Dimensions of segmental variability: Interaction of prosody and surprisal in six languages, *Frontiers in Communication 3*. 10.3389/fcomm.2018.00025.

[CR82] Marantz A. (2013). No escape from morphemes in morphological processing. Language and Cognitive Processes.

[CR83] Martinet A. (1965). La linguistique synchronique: études et recherches.

[CR84] Matthews P. H. (1991). Morphology.

[CR85] Mirkovic J., Seidenberg M. S., Joanisse M. F. (2011). Probabilistic nature of inflectional structure: Insights from a highly inflected language. Cognitive Science.

[CR86] Munson B. (2001). Phonological pattern frequency and speech production in adults and children. Journal of Speech, Language, and Hearing Research.

[CR87] OpenSubtitles (2013), https://www.opensubtitles.org.

[CR88] Pinker S. (1997). Words and rules in the human brain. Nature.

[CR89] Pinker S. (1999). Words and rules: The ingredients of language.

[CR90] Plag I., Homann J., Kunter G. (2017). Homophony and morphology: The acoustics of word-final S in English. Journal of Linguistics.

[CR91] Podlubny R., Geeraert K., Tucker B. (2015). It’s all about, like, acoustics. Proceedings of the ICPHS IIXX.

[CR92] Priva, U. C., & Jaeger, T. F. (2018). The interdependence of frequency, predictability, and informativity in the segmental domain. *Linguistics Vanguard, 4*(s2).

[CR93] Pylkkänen L., Feintuch S., Hopkins E., Marantz A. (2004). Neural correlates of the effects of morphological family frequency and family size: an MEG study. Cognition.

[CR94] Ramscar M., Port R. F. (2016). How spoken languages work in the absence of an inventory of discrete units. Language Sciences.

[CR95] Ramscar M., Yarlett D. (2007). Linguistic self-correction in the absence of feedback: a new approach to the logical problem of language acquisition. Cognitive Science.

[CR96] Ramscar M., Yarlett D., Dye M., Denny K., Thorpe K. (2010). The effects of feature-label-order and their implications for symbolic learning. Cognitive Science.

[CR97] Ramscar M., Dye M., Klein J. (2013). Children value informativity over logic in word learning. Psychological Science.

[CR98] Ramscar M., Dye M., McCauley S. (2013). Error and expectation in language learning: The curious absence of ‘mouses’ in adult speech. Language.

[CR99] Rastle K., Davis M. H., New B. (2004). The broth in my brother’s brothel: Morpho-orthographic segmentation in visual word recognition. Psychonomic Bulletin & Review.

[CR100] Roelofs A. (1997). Morpheme frequency in speech production: Testing WEAVER. Yearbook of morphology 1996.

[CR101] Roelofs A. (1997). The WEAVER model of word-form encoding in speech production. Cognition.

[CR102] Rumelhart D. E., McClelland J. L., Altmann G. T. M. (1986). On learning the past tenses of English verbs. Psycholinguistics.

[CR103] de Saussure F. (1916). Course de linguistique générale.

[CR104] Scarborough R. (2003). Lexical confusability and degree of coarticulation. Annual Meeting of the Berkeley Linguistics Society.

[CR105] Schmidtke, D., Matsuki, K., & Kuperman, V. (2017). Surviving blind decomposition: a distributional analysis of the time course of complex word recognition. *Journal of Experimental Psychology: Learning, Memory and Cognition.* 10.1037/xlm0000411 PMC5659973 28447810

[CR106] Schmidtke D., Gagné C. L., Kuperman V., Spalding T. L., Tucker B. V. (2018). Conceptual relations compete during auditory and visual compound word recognition. Language, Cognition and Neuroscience.

[CR107] Schriefers H., Meyer A. S., Levelt W. J. M. (1990). Exploring the time course of lexical access in language production: Picture-word interference studies. Journal of Memory and Language.

[CR108] Schulz E., Oh Y. M., Malisz Z., Andreeva B., Möbius B. (2016). Impact of prosodic structure and information density on vowel space size. Speech prosody 2016, Boston.

[CR109] Schwartz M. F., Brecher A. (2000). A model-driven analysis of severity, response characteristics, and partial recovery in aphasics’ picture naming. Brain and Language.

[CR110] Schwartz M. F., Dell G. S., Martin N., Gahl S., Sobel P. (2006). A case-series test of the interactive two-step model of lexical access: Evidence from picture naming. Journal of Memory and Language.

[CR111] Seyfarth S., Garellek M., Gillingham G., Ackerman F., Malouf R. (2018). Acoustic differences in morphologically-distinct homophones. Language, Cognition and Neuroscience.

[CR112] Shields L. W., Balota D. A. (1991). Repetition and associative context effects in speech production. Language and Speech.

[CR113] Sims, M. N. (2016). *The role of acoustic detail in the production and processing of vowels in spontaneous speech*. PhD Thesis, University of Alberta.

[CR114] Smolka E., Komlosi S., Rösler F. (2009). When semantics means less than morphology: The processing of German prefixed verbs. Language and Cognitive Processes.

[CR115] Solomyak O., Marantz A. (2010). Evidence for early morphological decomposition in visual word recognition. Journal of Cognitive Neuroscience.

[CR116] Sosnik R., Hauptmann B., Karni A., Flash T. (2004). When practice leads to co-articulation: the evolution of geometrically defined movement primitives. Experimental Brain Research.

[CR117] Stump G. T. (2001). Inflectional morphology: A theory of paradigm structure.

[CR118] Team R. D. C. (2018). R: A language and environment for statistical computing.

[CR119] Tiede M., Mooshammer C., Goldstein L., Shattuck-Hufnagel S., Perkell J. (2011). Motor learning of articulator trajectories in production of novel utterances. Proceedings of the ICPHS XVII, ICPHS.

[CR120] Tomaschek F., Wieling M., Arnold D., Baayen R. H. (2013). Word frequency, vowel length and vowel quality in speech production: An EMA study of the importance of experience. Proceedings of the interspeech.

[CR121] Tomaschek F., Arnold D., Broeker F., Baayen R. H. (2018). Lexical frequency co-determines the speed-curvature relation in articulation. Journal of Phonetics.

[CR122] Tomaschek F., Hendrix P., Baayen R. H. (2018). Strategies for managing collinearity in multivariate linguistic data. Journal of Phonetics.

[CR123] Tomaschek, F., Tucker, B. V., Fasiolo, M., & Baayen, R. H. (2018c). Practice makes perfect: the consequences of lexical proficiency for articulation. *Linguistics Vanguard*, *4*(s2).

[CR124] Tomaschek, F., Plag, I., Ernestus, M., & Baayen, R. H. (2019). Phonetic effects of morphology and context: Modeling the duration of word-final S in English with naive discriminative learning. *Journal of Linguistics*, 1–39.

[CR125] Tomaschek, F., Arnold, D., Sering, K., van Rij, J., Tucker, B. V., & Ramscar, M. (2020). Articulatory variability is reduced by repetition and predictability. *Language and Speech*, 1–27. 10.1177/0023830920948552 32811294

[CR126] Tucker, B. V., Sims, M., & Baayen, R. H. (2019). *Opposing forces on acoustic duration*. Technical report. Publisher: PsyArXiv. psyarxiv.com/jc97w.

[CR127] Turk A., Shattuck-Hufnagel S. (2020). Speech timing.

[CR128] van Rij J., Wieling M., Baayen R. H., van Rijn H. (2015). Itsadug: interpreting time series, autocorrelated data using GAMMs.

[CR129] Wieling M., Tomaschek F., Arnold D., Tiede M., Bröker F., Thiele S., Wood S. N., Baayen R. H. (2016). Investigating dialectal differences using articulography. Journal of Phonetics.

[CR130] Wittgenstein L. (1922). Tractatus logico-philosophicus.

[CR131] Wood S. N. (2006). Generalized additive models.

[CR132] Wood S. N. (2011). Fast stable restricted maximum likelihood and marginal likelihood estimation of semiparametric generalized linear models. Journal of the Royal Statistical Society (B).

[CR133] Wood S. N. (2013). On p-values for smooth components of an extended generalized additive model. Biometrika.

[CR134] Wood S. N. (2013). A simple test for random effects in regression models. Biometrika.

[CR135] Wright R., Local J., Ogden R., Temple R. (2004). Factors of lexical competition in vowel articulation. Phonetic interpretation.

[CR136] Yuan J., Liberman M. (2008). Speaker identification on the SCOTUS corpus. Journal of the Acoustical Society of America.

[CR137] Zipf G. (1949). Human behavior and the principle of least effort.

